# Complete genome sequence of *Methylomonas* sp. UP202 isolated from an urban waterway sediment

**DOI:** 10.1128/MRA.00633-23

**Published:** 2023-11-20

**Authors:** Beng-Soon Teh, Yiik-Siang Hii, Jamie Hinks, Mohd Firdaus Abdul-Wahab, Sanjay Swarup

**Affiliations:** 1 NUS Environmental Research Institute, National University of Singapore, Singapore, Singapore; 2 Singapore Centre for Environmental Life Sciences Engineering, Nanyang Technological University, Singapore, Singapore; 3 Department of Biosciences, Faculty of Science, Universiti Teknologi Malaysia, Johor Bahru, Johor, Malaysia; 4 Singapore Centre for Environmental Life Sciences Engineering, National University of Singapore, Singapore, Singapore; 5 Department of Biological Sciences, National University of Singapore, Singapore, Singapore; University of Delaware College of Engineering, Newark, Delaware, USA

**Keywords:** *Methylomonas*, methanotroph, whole genome sequencing, canal sediment

## Abstract

We report the complete genome sequence of *Methylomonas* sp. UP202 isolated from an urban waterway sediment in Singapore. The genome contains genes involved in methane, methanol, formaldehyde, and formate oxidation. It also contains genes utilizing various nitrogen sources such as nitrogen, nitrate, nitrite, urea, and ammonium.

## ANNOUNCEMENT

Methanotrophs offer various industrial applications (1–5). Here, we sequenced the genome of *Methylomonas* sp. UP202 to uncover its metabolic potential. An aerobic methanotroph *Methylomonas* sp. UP202 was isolated from the canal sediment at the Ulu Pandan Waterway in Singapore (1.3188° N; 103.7708° E). Sediment was added to ammonium mineral salt (AMS) (6) broth in a serum bottle supplemented with methane and air (20%:80%, vol/vol) and incubated at 30°C for a week. After several subcultures, colonies were selected on AMS agar with methane in Oxoid AnaeroJar. The purity of isolates was confirmed through phase-contrast microscopy and streaking on 10% TSA, with no growth indicating purity (7). Genomic DNA was extracted from 2 mL pure cultures grown in AMS broth using the Wizard Genomic DNA Purification Kit (Promega) following the manufacturer’s protocol for Gram-negative bacteria.

Library preparation for Illumina sequencing was performed according to Illumina’s TruSeq Nano DNA Sample Preparation protocol. The samples were sheared on a Covaris E220 to ~550 bp, following the manufacturer’s recommendation, and uniquely tagged with Illumina’s TruSeq HT DNA dual barcodes to enable sample pooling for sequencing. The libraries were sequenced on the Illumina MiSeq platform at a read length of 300 bp paired-end (Illumina, Inc.). A total of 4,354,004 paired-end reads were obtained for the MiSeq data. Oxford Nanopore (ONT) libraries were prepared following the manufacturer’s instructions for the SQK-LSK110 Genomic DNA by ligation library prep kit (Oxford Nanopore Technologies), together with the NEBNext Companion Module for ONT Ligation Sequencing (New England Biolabs). The library was loaded on a Flongle R9.4.1 flowcell using the Flongle adapter attached to a GridION Mk1 device and sequenced for 24 h on MinKNOW (v.22.08.9). The raw ONT data were basecalled using Guppy (v.6.2.1) and resulted in 134,811 ONT reads with an *N*_50_ value of 7,939 bp. Adapter sequences of short reads were removed using Cutadapt (v.3.5) (8), and low-quality reads were removed using BBDuk function in BBTools (v.37.99) (https://sourceforge.net/projects/bbmap/). The adapters of ONT long reads were trimmed using Porechop (v.0.2.3) (9), and the reads were quality filtered and trimmed using Filtlong (v.0.2.0) (https://github.com/rrwick/Filtlong). Short- and long-read data were hybrid assembled using Unicycler (v.0.4.8) (10). Platon (v.1.6) (11) was used for plasmid detection in the assembled genome. Average nucleotide identity (ANI) was determined using Pyani (v.0.2.11) (12). The assembled genome was annotated using the NCBI Prokaryotic Genome Annotation Pipeline (v.6.5) (13). The quality of hybrid assembly was evaluated using QUAST (v.5.2.0) (14).

The assembled reads resulted in two circular contigs, corresponding to a chromosome of 5,526,923 bp (GC content, 55.84%) and a plasmid of 148,958 bp (GC content, 52.27%). The genome of *Methylomonas* sp. UP202 contains 4,924 protein-coding genes, 9 rRNA operons (5S, 16S, and 23S), and 47 tRNAs. The ANI value between *Methylomonas* sp. UP202 and *Methylomonas* sp. LWB was ~97%. Enzymes such as methane monooxygenases (pMMO and sMMO), methanol dehydrogenases, and formate dehydrogenases were found in the genome. Gene clusters involved in nitrogen fixation (*nif*), urease, urea transporters, ammonium, nitrate, and nitrite reductases were present.

## Data Availability

The complete genome sequence of *Methylomonas* sp. UP202 has been deposited in GenBank under the accession numbers CP123897 and CP123898. Raw reads were submitted to the NCBI SRA under the accession numbers SRR24223047 and SRR24223048. The BioProject accession number is PRJNA954998 and the BioSample accession number is SAMN34162754.
